# Navigating the challenges of bicuspid aortic valve-aortopathy

**DOI:** 10.21542/gcsp.2023.27

**Published:** 2023-09-30

**Authors:** Sai Gautham Kanagala, Aanchal Sawhney, Kinna Parikh, Vasu Gupta, Talha Mahmood, FNU Anamika, Rohit Jain, Nikita Garg

**Affiliations:** 1 Osmania Medical College, Hyderabad, India; 2 Department of Internal Medicine, Crozer Chester Medical Center, Pennsylvania, USA; 3 GMERS Medical College, Gandhinagar, India; 4 Dayanand Medical College and Hospital, Ludhiana, India; 5 Florida International University, Florida, USA; 6 University College of Medical Sciences, New Delhi, India; 7 Penn State Health Milton S Hershey Medical Center, Hershey, Pennsylvania, USA; 8 Department of Pediatrics, Southern Illinois University School of Medicine, Springfield, Illinois, USA

## Abstract

Bicuspid aortic valve (BAV) is a congenital heart defect that affects 0.5–2% of the general population with familial predominance. The modifications in hemodynamics and structure change at cellular level contribute to the dilation of aorta, resulting in bicuspid aortopathy, which can result in catastrophic aortic events. The American Heart Association recommends screening first-degree relatives of patients with bicuspid aortic valve and aortic root disease. BAV may or may not be associated with a syndrome, with the non-syndromic variety having a higher chance of predisposition to congenital and vascular abnormalities. Many genes have been implicated in the etiology of non-syndromic aortic aneurysm such as ACTA2, MYH11, FLNA, and SMAD3. Common diagnostic modalities include transthoracic echocardiography (TTE), transesophageal echocardiography (TEE), multi system computer tomography (MSCT), and cardiac MRI. Medical management reduces the rate of disease progression and surgical management is indicated based on the diameter of the ascending aorta, which differs in American and European guidelines. Our article aims to explore the current understanding of the pathophysiology, clinical aspects, and surgical management of bicuspid aortic valve disease. Additionally, we have included a discussion on the management of this condition in special populations, such as athletes and pregnant women, who require distinct treatment recommendations.

## Introduction

Bicuspid aortic valve (BAV) is the most common congenital cardiac valvular abnormality, with a prevalence among the general population of between 0.5 and 2% and a 3:1 male-to-female ratio^1^. The prevalence of BAV in first-degree family members is 10-fold higher than in the general population.

BAV describes an aortic valve with two leaflets, instead of the normal three, resulting from abnormal fusion during embryonic development^2^. BAV is often identified incidentally in otherwise healthy individuals, but it is associated with serious long-term health risks. Patients with BAV have complications occurring a decade earlier in life than in patients with a tricuspid aortic valve (TAV), such as aortic valve disease (stenosis and/or regurgitation), infective endocarditis and thrombus formation and is associated with ascending aortic aneurysm (AA) and dissection^5^.

BAV is primarily inherited in an autosomal dominant pattern with incomplete penetrance and variable expressivity. It is also associated with syndromes such as Turner syndrome and Williams syndrome, connective tissue disorders like Marfan syndrome and Loeys-Dietz syndrome, and the vascular Ehlers-Danlos syndromes^6^.

In non-syndromic cases, the occurrence of BAV is best explained by a complex genetic architecture involving many different interacting genes. People with BAV who do not have syndromic features have higher chances of predisposition to other congenital heart and vascular abnormalities like coarctation of aorta (7%), patent ductus arteriosus (8.5%), mitral valve abnormalities (11%), ventricular septal defects (14%) and thoracic aortic aneurysm (50%)^7^.

According to familial clustering studies, BAV is heritable, with an incidence of 9% in first-degree relatives of patients with BAV and up to 24% in families with more than one afflicted member. Thus, the association of several alarming outcomes, such as bacterial infective endocarditis (IE) and aortic dissection, with the high frequency of BAV in people emphasizes the significance of screening for BAV.

The American Heart Association (AHA) currently recommends screening first-degree relatives of patients with bicuspid aortic valve and aortic root disease^8^. Transthoracic echocardiography is the diagnostic tool of choice for BAV, but transesophageal and/or 3-dimensional echocardiography is sometimes necessary to confirm the diagnosis^9^.

BAV is a highly heterogeneous congenital heart disease that is characterized by malformations of the aortic valve associated with genetic syndromes, aortopathy, and other congenital heart defects. Aortic dilation, most commonly being the thoracic aorta dilation is the key feature of bicuspid aortopathy^11^.

In 1928, the association between aortic diseases and BAV was first established by Abbott^12^. In contrast to aortic aneurysm (AA) formation in patients with a normal tricuspid aortic valve (TAV), dilatation in patients with a BAV starts much earlier (by at least 10-15 years) and progresses in a faster and continuous manner^13,14^. The development and progression of AA are attributed to different genetic, haemodynamic, and cardiovascular risk factors. This review article will discuss our current knowledge of pathophysiology, clinical aspects, and surgical management of bicuspid aortic valve disease.

## Mechanism of BAV associated aortopathy

### Morphology

The International Consensus Classification and Nomenclature for the congenital bicuspid aortic valve condition recognizes 3 types of bicuspid valves: 1. The fused type (right-left cusp fusion, right-non-coronary cusp fusion, and left-non-coronary cusp fusion phenotypes); 2. The 2-sinus type (latero-lateral and antero-posterior phenotypes); and 3. The partial-fusion (forme fruste) type^3^.

These fusion patterns of BAV have been shown to result in aberrant blood flow dynamics through the aortic valve^4^. Ascending aorta dilation (AAD) refers to enlargement or widening of aorta, which is the initial segment coming out of the aorta and can be classified into aortic root AAD or tubular AAD.

Aortic root AAD is located above the sinotubular junction (STJ, i.e the junction between the aorta and the coronary arteries) and tubular AAD is located below the STJ, based on embryonic origin of the tissue and functional characterization of valve morphology and disease^3^. Only weak association exists between the BAV type morphology (right-left *vs.* right-non-coronary fusion) and aortic phenotype (root *vs.* tubular dilation)^15^.

### Etiology

The etiology of AAD and dissection in association with BAV is due to increased wall stress associated with structural and functional abnormalities in the aortic wall^4^. Although stenosis was considered as the cause of eccentric blood flow causing altered shear stress, abnormal flow patterns exist in the absence of stenosis in patients with BAV with AAD^16^.

Fusion of right and noncoronary cusps is associated with dilation of tubular aorta, but not exclusively^17,18^. Although hemodynamics play an important role in the etiology, clinical data states a lower incidence of aortic events such as surgery or AAD after resection of stenotic aortic valve. The factor favoring genetic etiology is the continued dilation of aorta after curative valve replacement^19,20^. Imaging data, including use of magnetic resonance imaging which measures 3-dimensional blood flow through the valve and aorta, supports the hemodynamic etiology of tubular AAD^21^.

Different age groups and body habitus have limited the ability to identify biological mechanisms of AAD across a wide phenotypic spectrum, such as root aneurysm *vs.* ascending aortic aneurysm and dilated *vs.* normal aorta^4^.

Histologically, BAV aortopathy shows non-inflammatory loss of smooth muscle cells (SMCs), with multifocal apoptosis and medial degeneration. Similar to Marfan syndrome, BAV associated aortic tissues have lower fibrillin content and increased TGF- *β*1 levels^22,23^.

Despite evidence of shear stress in the tubular ascending aorta altered by BAV, leaflet fusion is not the sole predictive factor as the presence of age and clinical characteristics also play an important role^24,25^. The theories supporting genetics as the cause of BAV aortopathy include gene pleiotropy, as the genes associated with BAV are also associated with AAD^4^.

### Genetic factors

BAV is inherited in an autosomal dominant pattern^26^. Studies have revealed a high incidence of familial clustering^27^. According to AHA, the genes linked with bicuspid aortic valve-associated ascending aortic aneurysm are *NOTCH1, TGFBR2, MAT2A, GATA5, SMAD6, LOX, ROBO4, TBX20,* XO and Xp^28^.

It has been noted that nonsyndromic AA is usually linked to genes like *ACTA2*, *MYH11*, *FLNA* and *SMAD3,* whereas syndromic AA is associated with *FBN1*, *TGFBR1* and *TGFBR2* ^7^. However, the genetic basis of the BAV-associated aortopathy is unclear to date. The genetic linkage analyses revealed that the familial BAV as well as the associated thoracic aneurysm is linked to the chromosome 15q25–26^29^; the relevant genes however remain unknown.

Many genes have been implicated in the etiology of non-syndromic aortic aneurysm such as *ACTA2, MYH11, FLNA,* and *SMAD3*. Likewise, other genes such as *FBN1* and transforming growth factor beta receptor (*TGFBR1* and *TGFBR2*) have been implicated in the development of syndromic AA, but none have been proven to be conclusive in causing BAV aortopathy^7^. The potential role of microRNAs (miRNAs) as essential epigenetic components in several cellular processes related to BAV aortopathy has attracted attention in recent years^10^.

### BAV and the aorta

Along with genetic factors, altered flow within the ascending aorta due to valve malformations also plays a central role in premature dilation of an already weakened aortic wall. Different phenotypes of BAV cause characteristic hemodynamic patterns resulting in ‘wall shear stress’ (WSS).

An experimental *in vitro* study by Atkins et al., used fluid structure interaction (FSI) to assess the difference in character of the convexity of ascending aorta at the cellular level, based on the exposure to BAV *vs* tricuspid aortic valve (TAV) AA WSS for 48 h^26,29^.

Results of the FSI study revealed the existence of larger and more unidirectional WSS in BAV AA compared to TAV AA convexity. In comparison to TAV AA WSS, normal aortic tissue, when exposed to BAV AA WSS treatment, revealed an increase in MMP-2, MMP-9 expression and MMP-2 activity, but similar fibrillin-1 content and microfibril organization, confirming the aortic sensitivity to WSS abnormalities and demonstrating the potential of BAV hemodynamic stress to focally mediate aortic medial degeneration^29^. However no remodelling changes were observed in the concave wall of ascending aorta^30^. Patients with non-dilated type-I BAV AA with left–right-coronary cusp fusion achieved most significant abnormality on the wall convexity, measured in terms of oscillatory shear index, indicating the original of anomalies valvular rather than aortic dilation^31^.

A study conducted by Mahadevia et al. showed that the presence and the type of BAV fusion were associated with changes in regional WSS distribution, systolic outflow asymmetry, and expression of BAV aortopathy. In patients with right- and non-coronary cusp fusion, the right-posterior aortic wall is exposed to the highest WSS, and it is exactly these kinds of patients who often suffer from dilatation of the entire aorta and the aortic arch, or dilatation of the aortic root alone.

In contrast, patients with a left–right coronary cusp fusion have the maximum WSS at the right-anterior wall of the aorta and suffer from an isolated dilatation of the ascending aorta^32^. However, Jackson *et al.* concluded that there is no pattern in aortic dilation in 300 BAV patients undergoing open heart surgery related to leaflet morphology^33^. Additionally, recent studies have shown that structural changes occur at cellular levels independent of hemodynamic lesions, as it has been observed that the degree of stenosis was not always proportional to that of turbulence^34^.

BAV exhibits premature cystic medial degeneration in around half of BAV aortas^35^. The thoracic aorta demonstrates reduced fibrillin-1 content, elastin fragmentation, and apoptosis independent of valve function^36–38^. Decreased fibrillin-1 leads to smooth muscle cell detachment, matrix disruption, and cell death^36^. Similar structural abnormalities are also seen in the pulmonary trunk, but are clinically insignificant^37^.

## Diagnosis

The diagnostic approach for the initial assessment of BAV involves a transthoracic echocardiography (TTE), which is not only useful in evaluating the valvulopathy but is useful in the assessment of the thoracic aorta^39^. Four commonly used diagnostic imaging modalities are TTE, transesophageal echocardiography (TEE), multi-system computer tomography (MSCT), and cardiac MRI (Table 1)^40^.

**Table 1:** Diagnostic modalities used for the diagnosis of aortopathy in patients with BAV.

Diagnostic modality	Indication	Advantages	Disadvantages
TTE (Transthoracic Echocardiography)	1st step in diagnosis	Cost-effective No radiation exposure Evaluate valvulopathy and thoracic aorta	Reader dependent Limited by imaging window
TEE (Transesophageal Echocardiography)	Difficult TTE study	No radiation exposure	Need conscious sedation Invasive Able to visualize coronary artery and aortic arch but not fully
MSCT (Multi System Computer Tomography)	If Aortic diameter > 40 mm on TTE	Short scan time Calcification well visualized High spatial resolution No limitation by imaging window	Use of contrast Limited availability Radiation exposure Aortic valve or LV function not well visualized.
Cardiac MRI (Magnetic Resonance Imaging)	If Aortic diameter > 40 mm on TTE In pregnant patients Contraindication to radiation	No exposure to radiation Hemodynamic assessment Multiple parameter assessment- LV dimension and function	Limited availability C/I in patients with implanted cardiac devices Long scanning time Claustrophobic patients will require sedation

TTE is the imaging technique of choice for identifying BAV, valve morphotype, and for evaluating the severity of valvular dysfunction and guiding appropriate management decisions. However, it can be less accurate in assessing the aortic root and proximal ascending aorta, and visualization of the mid-distal ascending aorta and the arch may be challenging in some adults, where cardiac magnetic resonance and computed tomography, using multiplanar reconstructions, are better at assessing aortic diameters^41^.

In recent years, there have been advancements in the identification of chemical and imaging markers that can aid in the detection of patients at risk of aortic aneurysm and dissection. Various molecular markers of aortic wall dysfunction, such as soluble receptor for advanced glycation end product (RAGE), sphingomyelins, matrix metalloproteinases (MMP-2, MMP-8), tissue inhibitors of metalloproteinases (TIMP-1, TIMP-3, TIMP-4), alpha-1-antitrypsin, and endothelial microparticles (PCAM (+) EMPs), have shown promise in risk stratification^6,42–44^.

Furthermore NT-pro BNP levels may be elevated in individuals with BAV who have developed complications such as aortic stenosis or aortic dilation (aortopathy)^45^. These markers can provide valuable insights into the underlying pathophysiology of aortopathy and aid in the diagnosis and management of BAV-aortopathy patients. Additionally, the use of 4-dimensional flow MRI has allowed for the identification of specific imaging phenotypes that can enhance diagnostic accuracy. By combining molecular markers of aortic wall dysfunction, imaging phenotypes, and genetic risk factors, clinicians can achieve greater precision in assessing the severity of aortic valve disease and identifying patients at higher risk for adverse outcomes^46^.

## Management

The aortopathy associated with BAV disease can be managed medically or surgically depending on characteristics like size and the rate of increase of the aortic root dilation, family history of aortic dissection, presence of concomitant valve repair or replacement, aortic root phenotype and the new addition to the indications include the ratio of aortic root dilation to the height^44^.

Regular monitoring, genetic counseling, and a heart-healthy lifestyle are essential for managing aortopathy in BAV disease. Patient education, multidisciplinary care, and research contribute to effective management. However, limited outcome data exist regarding the impact of these modifications^47^.

### Medical intervention

Medical interventions to slow the rate of disease progression include beta-blockers, angiotensin-converting enzyme inhibitors (ACEi), and angiotensin receptor blockers^6^.

Beta-blockers are commonly used to decrease the stretch on the aortic wall, thereby slowing the progression of aortic root dilation. Recent guidelines recommend using the maximum tolerated dose of beta-blockers^48^. Angiotensin-converting enzyme inhibitors (ACEi) and angiotensin receptor blockers (ARBs) have also been investigated as they can inhibit the signaling pathways involved in aortic wall remodeling. ARBs such as losartan inhibit the angiotensin I mediated TGF- *β* signaling pathway that is profibrotic and mediates apoptosis, elastin degradation which AT II attempts to repair through fibrosis^49^. It is important to note that statins have shown to decrease mortality in abdominal aortic aneurysm (AA) but not in ascending AA^50^.

### Surgical intervention

Surgical intervention is recommended when certain criteria are met. The American Heart Association (AHA) recommends surgical surgery when the diameter of the aortic sinuses and/or ascending aorta exceeds 5.5 cm. Furthermore, if a patient with BAV has indications for surgical aortic valve replacement (SAVR) and the diameter of the aortic sinuses and/or ascending aorta exceeds 4.5 cm, surgery at a Comprehensive Valve Centre may be deemed appropriate^51^. The valve should be replaced in bicuspid aortic valve (BAV) patients with aortic insufficiency, symptomatic severe stenosis, and asymptomatic patients with aortic insufficiency and left ventricular dilation or dysfunction^52^.

1. In cases where intervention is limited to the aortic valve, particularly in individuals with an aortic diameter less than 4.5 cm, various alternatives are available. These include mechanical valve replacement, bioprosthetic valve replacement, pulmonary autograft (Ross operation), or aortic valve repair^45^.
2. In patients with BAV disease with aortic root dilation, the operation of choice has generally been aortic root replacement with a valved conduit including coronary artery reimplantation, also known as a Bentall procedure^53^.
3. Furthermore, for patients in need of root but not valve intervention, the surgical options include a valve-sparing aortic root procedure^45^.

A comprehensive review of surgical management, along with a classification of recommendations, is presented in Table 2. This table provides a comparison between the guidelines provided by the American and European medical societies, offering detailed information on the surgical approaches recommended for managing aortopathy associated with BAV disease.

**Table 2:** Indications for surgical management of aortic repair as recommended by American and European guidelines^28, 54–56^.

Recommendation	American Guidelines	European Guidelines
Beta-blockers for BAV and dilated aortic root > 40 mm	No class of recommendation, Only in text (VHD 2014)	IIb C (AD 2014)
Surgical intervention in BAV without additional risk factors	Maximal aortic diameter ≥ 55 mm (IB) (GC 2016)	Maximal aortic diameter ≥ 55 mm (IC)
Surgery indicated in patients with BAV and aortic aneurysm with risk factors.	Maximal aortic diameter ≥ 50 mm (IIaB) (GC 2016)	Maximal aortic diameter ≥ 55 mm (IC)
ESC- family history of aortic dissection (AD), aortic size increase > 3 mm/year, aortic coarctation, hypertension		
ACC/AHA- family history of AD, aortic coarctation, aortic root phenotype increase > 3 mm/year		
When to consider patients for concomitant aortic root repair of aortic replacement if they are undergoing aortic valve repair or replacement with BAV	Maximal aortic diameter ≥ 45 mm (IIaB) (GC 2016)	Maximal aortic diameter ≥ 45 mm (IIaB) (GC 2016)
Maximal aortic root or ascending aortic diameter to height ratio ≥ 10 cm^2^/m should undergo elective prophylactic aortic repair	IIa recommendation	

## Management in special populations

### Children

For children, annular dilation has been identified as an independent risk factor for the progression of aortic regurgitation in BAV, and an aortic dilation of 25-27 mm is considered as the cut-off for aortic annuloplasty^57^. Current guidelines recommend replacement of ascending aorta in cases of aortic replacement in cases of a diameter > 45 mm if a concomitant aortic valve surgery is planned.

### Athletes

Braverman et al. recommend that all athletes with BAV can participate in competitive sports if ascending aorta and aortic root dilation is less than 2 standard deviations from the mean and less than 40 mm in adults^58^. For recommendations regarding sports participation, Task Force 5 on Valvular Heart Disease Left Ventricle End Systolic Diameter identified left ventricular ejection fraction, left ventricle end diastolic diameter and valve area, and severity of valvular involvement as important factors in determining suitability. They recommend that athletes with BAV, AR and aortic dimensions of 41 to 45 mm can participate in sports with low risk of bodily contact (Class IIb; Level of Evidence C). The 2015 ACC/AHA guidelines for patients with BAV and cardiovascular abnormalities recommend annual TTE or MRI angiography if the aortic dilation is in the range of 40-42 mm in men and 36–39 mm in women (class I recommendation, level of evidence C)^59^.

### Pregnant females

WHO risk classification in pregnant patients is based on morbidity and mortality with aortic wall diameter as primary criterion. In patients with aorta <45 mm, intermediate risk of mortality is reported, in patients with aorta 45-50 mm, significantly increased risk of mortality is reported and in patients with aorta > 50 mm, pregnancy is contraindicated due to extremely high risk of maternal mortality^60^.

ESC guidelines for cardiovascular disease during pregnancy recommend a pre-pregnancy prophylactic surgery if aortic diameter is > 50 mm and depending upon the aortic diameter, TTE at 4-12 week intervals throughout the pregnancy and 6 months postpartum^61^.

### Endocarditis risk

The incidence of infective endocarditis (IE) in the bicuspid aortic valve population ranges from 10% to 30%. Twenty-five percent of IE cases occur in a bicuspid aortic valve^62^. Regarding antimicrobial prophylaxis, the most recent American Heart Association guidelines do not recommend its routine use prior to invasive dental procedures for normally functioning BAV or BAV with aortic stenosis or aortic regurgitation. However, if a patient has a history of prior endocarditis or has undergone aortic valve replacement or aortic valve repair, antibiotic prophylaxis is recommended^63^.

## Follow up

In patients with transthoracic aortic diameter > 40 mm measured on TTE, confirmation should be obtained with CT or MRI. If comparable and reproducible, TTE should be sufficient for follow-up measurements.

According to the ACC/AHA guidelines, the first degree relative of patients with a known diagnosis of BAV should be screened with TTE to look for the presence of a BAV or asymptomatic dilation of aortic sinus and ascending aorta (Class 2b recommendation)^43^.

In patients with BAV, if initial imaging did not reveal any aortic wall dilation, serial imaging can be repeated every 10-15 years and in case of dilation, annual imaging is warranted^5^. Post isolated aortic valve repair or replacement, annual surveillance is recommended to monitor progressive aortopathy, due to an increased risk of dissection or rupture after isolated valve surgery due to alterations in the hemodynamics. In patients with replacement or repair of ascending aorta, it is recommended repeat imaging with MRI or CT angiography at 3-5 year intervals to check for complications^44^.

## Conclusion and future work

Bicuspid aortic valve can cause a variety of pathologies, such as narrowing or a dilation of aortic valve orifice, leading to aortic stenosis or regurgitation respectively, or dilation of the aorta itself leading to an aneurysm which further increases the risk of an aortic dissection.

Screening using TTE is indicated in those with a first-degree relative suffering from BAV. Medical management involves lowering the blood pressure and stress on the aortic wall using beta-blockers and ACE inhibitors. Surgical intervention depends on the diameter of the ascending aorta as well as the rate of expansion and the presence of other risk factors. Surgical valve replacement is indicated when the diameter of the aorta is more than 5.5 cm. Our knowledge about the genetics, pathophysiology, and treatment for BAV has widened but there remain some important unanswered points regarding the surveillance and universal management guidelines. There is still little knowledge on the exact pathophysiology of BAV and further research is required in this field. Better diagnostic tools for early detection and management of BAV is required to prevent morbidity and mortality in patients with BAV.

## Abbreviations

Bicuspid aortic valve (BAV); tricuspid aortic valve (TAV); aortic aneurysm (AA); microRNAs (miRNAs); Transthoracic Echocardiography (TTE); Transesophageal Echocardiography (TEE); Multi System Computer Tomography (MSCT); Wall Shear Stress (WSS); Magnetic Resonance Imaging (MRI); angiotensin-converting enzyme inhibitors (ACEi); infective endocarditis (IE); transforming growth factor beta receptor (TGFBR); surgical aortic valve replacement (SAVR); American Heart Association (AHA).

## Author contribution statement

**Conceptualization**: Sai Gautham Kanagala, Aanchal Sawhney, Kinna Parikh.

**Supervision**: Rohit Jain, Nikita Garg.

**Writing - Original Draft Preparation**: Sai Gautham Kanagala, Aanchal Sawhney, Kinna Parikh, Vasu Gupta, Talha Mahmood.

**Writing - Review & Editing:** Sai Gautham Kanagala, FNU Anamika, Rohit Jain, Nikita Garg.
